# Green Synthesis: The Antibacterial and Photocatalytic Potential of Silver Nanoparticles Using Extract of *Teucrium stocksianum*

**DOI:** 10.3390/nano13081343

**Published:** 2023-04-12

**Authors:** Iqra Rehman, Humaira Yasmeen Gondal, Roshan Zamir, Sami A. Al-Hussain, Fozia Batool, Ali Irfan, Sobia Noreen, Taleeha Roheen, Muhammad Nisar, Magdi E. A. Zaki

**Affiliations:** 1 Institute of Chemistry, University of Sargodha, Sargodha 40100, Pakistan; iqrarehman0042@gmail.com (I.R.); zamirg786@gmail.com (R.Z.); fozia.batool@uos.edu.pk (F.B.); sobia.noreen@uos.edu.pk (S.N.); taleeha.rohin@uos.edu.pk (T.R.); nisarbutt88@gmail.com (M.N.); 2 Department of Chemistry, College of Science, Imam Mohammad Ibn Saud Islamic University (IMSIU), Riyadh 13623, Saudi Arabia; sahussain@imamu.edu.sa; 3 Department of Chemistry, Government College University Faisalabad, Faisalabad 38000, Pakistan; raialiirfan@gmail.com

**Keywords:** *Teucrium stocksianum*, silver nanoparticles, multi-drug-resistant pathogens, photolytic degradation

## Abstract

Green synthesis is one of the promising pathways for biologically active nanoscale materials. Herein, an eco-friendly synthesis of silver nanoparticles (SNPs) was carried out using an extract of *Teucrium stocksianum*. The biological reduction and size of NPS were optimized by controlling the physicochemical parameters such as concentration, temperature, and pH. A comparison of fresh and air-dried plant extracts was also undertaken to establish a reproducible methodology. The biosynthesized SNPs were characterized by UV–Vis spectroscopy, FT-IR, SEM, DLS, and XRD analyses. The prepared SNPs exhibited significant biological potential against multi-drug-resistant pathogenic strains. The results revealed that the biosynthesized SNPs exhibit high antimicrobial activity at low concentrations compared to the parent plant extract. Minimum inhibition concentration (MIC) values were found between 5.3 µg/mL to 9.7 µg/mL for the biosynthesized SNPs, whereas the aqueous extract of the plant showed many high values of MIC, i.e., between 69 and 98 µg/ML. Furthermore, the synthesized SNPs were found efficient in the photolytic degradation of methylene blue under sunlight.

## 1. Introduction

Nanotechnology has attracted the attention of scientists all around the world due to its wide applications in various fields. Generally, the term nanotechnology has been applied to particles ranging in size from 1 to 100 nm [1]. Properties of nanoparticles like shape, size, and large surface area-to-volume ratio make them useful in various fields. These fields include the pharmaceutical, cosmetics, and food industries, electronics, energy saving, textiles, etc. [2]. Nanomedicine is an interdisciplinary field that combines the knowledge of chemistry, engineering, biology, and medicines, providing an effective tool for the prevention and treatment of different diseases [3]. Nanotechnology has explored new advances in the medicinal field like sensing [4], imaging [5], gene delivery [6], and target drug delivery [7].Nanoparticles of ceramics, metals, and polymers can combat human pathogens [8,9]. Currently, several nano-based medicines such as Caelyx, Dolix, Abraxane, and Transdrug are approved by the FDA [10]; lipid nano-emulsions, graphene, nanosphere, and carbon nanotubes have been used for gene/drug delivery [11], and silica-based nanoparticles are reported as a therapeutic and bioimaging agent [12].

Different conventional methods for the synthesis of nanoparticles have many associated disadvantages. For instance, chemical methods involve the use of hazardous, toxic, and nondegradable chemicals and solvents as reducing and capping agents. Similarly, the physical method requires complex and expensive instruments and high temperatures or pressure [13,14]. Therefore, green and environment-friendly methods for the synthesis of NPs are highly demanding [15]. In this context, the synthesis of metal nanoparticles using plant extracts is gaining popularity due to their low cost and easy availability. Moreover, plant extract can play a dual role as a reducing agent as well as a capping agent. The biological activities of nanoparticles are greatly influenced by their size, shape, and morphology. We can control the size, shape, and morphology of the nanoparticles by changing factors like pH, extract concentration, and temperature of the reaction media [16]. On the other hand, plant-based nanoparticles possess an additional layer of biologically active secondary metabolites which can enhance the effectiveness of metallic nanoparticles [17]. Plant-mediated silver nanoparticles are widely reported as a potential pharmaceutical agent to cure various alignments like cancer, HIV, tumors, hepatitis, and many other diseases [18].

As anti-bacterial agents, AgNPs were implemented in numerous applications ranging from disinfecting medical devices, home appliances, and water treatment and in textile industry [19,20]. Moreover, this inspired the textile industry to use AgNPs in numerous textile goods. The practical applications of disinfectants based on AgNPs have attained much applications due to their use in daily life. They found applications in silver-based air\water filters. Moreover, this inspired the textile industry to use AgNPs in numerous textile goods [21,22]. The anti-bacterial activities of silver nanoparticles are reported against *Escherichia coli*. When E. coli are treated with AgNPs, pits are formed in the cell wall of the bacteria leading to cell death. Similar [23] results have been reported by Ref. [24]. In addition to this, AgNPs have demonstrated efficient inhibitory activities against the human immunodeficiency virus (HIV) and hepatitis B virus (HBV) [25]. AgNPs also inhibit the growth of many gram-negative strains like *Acinetobacter* [26], Salmonella [27], and *Pseudomonas* [28]. The effectiveness of AgNPs was also reported for gram-positive bacteria including *Bacillus* [29], *Enterococcus* [30], and *Streptococcus* [31].

The AgNPs synthesized from leaf extracts of *Prosopis juliflora* were found workable in wastewater treatment [32]. Similarly, Terminalia bellirica fruit extract-mediated silver nanoparticles were effective in treating the water from the textiles industry [33] *Acacianilotica* pod-mediated silver nanoparticles changed the glassy carbon conductor to express larger catalytic activity on the reduction of benzyl chloride compared to those of the glassy carbon and metallic Ag electrode [34]. Photocatalytic degradation of methyl orange was measured spectrophotometrically using *Ulva lactuca* and synthesized AgNPs as nano catalysts under visible light illumination [35]. The manufactured AgNPs using *Gloriosa superb* plant extract act through the electron relay effect and influence the degradation of methylene blue at the end of 30 min [36] AgNPs were synthesized using *A. dubius* fabricated on cotton cloth, and the perspiration pad samples express high resistance towards *Corynebacterium*, a sweat microorganism [37]. The antibacterial drug activity of gauze fabric discs incorporated with AgNPs, made from thein experienced mature thalli of *Anthoceros,* exhibits antimicrobial activity against *Pseudomonas aeruginosa* [38]. The effectiveness of silver nanoparticles against coronavirus has also been recently reported. Cur-Ag nanoparticles were evaluated with a surface corona of isoniazid, tyrosine, and quercetin. Among various nanoparticles, Cur-AgQrc showed the highest radical scavenging activity and a positive effect on cell viability and cell proliferation [39]. C-phycocyanin-primed silver nanohybrids (AgcPCNP) were found effective in the development of nanomedicines that aid in wound healing [40]. AgcPCNPs were found to be effective in dermal wound healing [41]. Similarly, surface functionalized AgcPCNP accelerated the fibroblast cell migration.

Pakistan is blessed with a wide variety of medicinally diverse flora. The genus Teucrium, which is the part of the Lamiaceae family, contains 340 species, with four species reported in Pakistan. The medicinal properties of various Teucrium species have been studied in literature, such as antioxidant, antibacterial, and antifungal activities [42]. *Teucrium stocksianum* is an aromatic, perennial herb found in hilly parts of Iran, the Middle East, and Pakistan. Ethanolic extracts of *Teucrium stocksianum* are reported with analgesic and anti-inflammatory activity that was traditionally used in rheumatism and other related diseases [43].The crude extract of *Teucrium stocksianum* and its fractions possess antioxidant, antileishmanial, bactericidal, and fungicidal antispasmodic activities [44]. The juice of *Teucrium stocksianum* extract is also used for the treatment of jaundice and as a blood purifier as well as a cooling agent in remote areas. Leaves were soaked in water overnight and the juice was given before breakfast to the patient withdiarrhea and abdominal pain. Boiled leaf extract is used to cure cough [45]. Different solvent extracts of the *Teucrium stocksianum* flower possess sufficient phytochemicals such as flavonoids, saponins, and phenolic compounds. These phyto-constituents demonstrate free radical scavenging and high antioxidant activities [46].

## 2. Materials and Methods

In the current study, washed and oven-dried glassware was used. Deionized water was used for the preparation of all solutions. AgNO_3_ (99.98%) from Merck, Darmstadt, Germany was used for the preparation of SNPs. UV–Vis analyses were performed on a UV-1700 spectrophotometer (Shimadzu, Koyoto, Japan). FT-IR spectra were recorded on IR Prestige-21 Perkin Elmer Nicolet 510P (Shimadzu, Koyoto, Japan). Scanning electron microscopy was performed on a scanning electron JEOL IT100LA (JEOL, Tokyo, Japan) for surface morphology, distribution, and size of NPS. Dynamic light scattering (DLS) was performed on Malvern zetasizerNanoZS, Worcestershire UK to determine particle size distribution and zeta potential. Powder X-ray diffraction measurements were carried out (over a range of 5–100°, 2ϴ) on Rigaku MiniFlex 600c, Tokyo, Japan. The aerial part of *Teucrium stocksianum* was collected from Makerwal, Mianwali, Pakistan.

### 2.1. Preparation of the Plant Extract

The plant material was washed with distilled water. Next, 10 g of fresh *Teucrium Stocksianum* ariel parts were soaked in 600 mL distilled water overnight with constant stirring. It was then filtered with filter paper. In the residue, 400 mL of water was further added; this was left for 12 h, and then filtered. The residue was discarded, and the filtrate was stored. The extract obtained was named TSFPE (*Teucrium stocksianum* fresh plant extract). The ariel parts of the plant were dried under shade. The air-dried plant was then ground with a grinder. Next, 5 g of the powdered plant was put into the beaker and soaked well in 500 mL water by continuous stirring for 12 h. The solution was filtered with filter paper. Then, 350 mL of water was added to the residue and soaked for 6 h by continuous stirring followed by filtration. The filtrate obtained was dried to get an extract named TSADE (*Teucrium stocksianum* air-dried extract). These two types of extract were prepared to determine the difference in the potential of fresh and dried plant parts for the synthesis of silver nanoparticles and their antibacterial activities.

### 2.2. Preliminary Phytochemical Screening of Teucrium stocksianum

The crude aqueous extract of *Teucrium stocksianum* was screened for saponins, alkaloids, tannins, waxes, sterols, terpenoids, and flavonoids by performing different identification tests using standard procedures. The aqueous extract of *Teucrium stocksianum* was collected in a test tube. Hager’s reagent (a saturated solution of picric acid) was added dropwise. Yellow-colored precipitates showed the presence of alkaloids in the plant extract. The presence of tannins was detected by adding a freshly prepared 5% FeCl_3_ solution to the aqueous plant extract. Light brown color was changed to dark green, confirming the presence of tannins. An aqueous solution of extract was well shaken and the appearance of foam indicated the presence of saponins. A test for sterols was performed by adding conc. H_2_SO_4_ to the chloroform solution of the plant extract. The appearance of a red-colored ring indicated the presence of sterols. On addition of the alcoholic alkali solution (0.5 M NaOH) to the plant extract, no saponification was observed, which indicated the absence of waxes in the *Teucrium stocksianum* extract. Following the addition of 2M NaOH solution to the aqueous extract of the plant, a yellow color appeared, which disappeared after the drop-wise addition of dil. HCl, indicating the presence of flavonoids in the plant extract.

### 2.3. General Procedure for the Preparation of Silver Nanoparticles Using Teucrium stocksianum

Silver nanoparticles were synthesized by adding 1 mL of stock solution of *Teucrium stocksianum* (50 mg/50 mL) extract to a 9 mL AgNO_3_ (5 mM) solution. The reduction of Ag^+^ to Ag° was indicated by a color change from watery yellow to dark brown. The colloidal mixture was centrifuged for 10 min at 1000 rpm followed by re-dispersing in deionized water, and the process was repeated three times to obtain the pure SNP powder, which was further dried in the hot air oven for 4 h. The effects of various factors like pH, concentration, and temperature were studied by monitoring the changes on a UV–Vis spectrophotometer.

#### 2.3.1. Effect of the Extract Concentration

Aqueous solutions of various concentrations (1 mg/1 mL to 5 mg/1 mL) were prepared for TSFPE and TSADE in separate flasks. The concentration of AgNO_3_ was kept constant at 5 mM. Results were recorded by the standard procedure discussed above.

#### 2.3.2. Effect of pH

The experiment was repeated by adjusting the pH of the reaction mixture (3, 7, and 9) by adding solutions of HCl and NaOH as required. Meanwhile, the concentration of plant extract and silver nitrate was kept constant.

#### 2.3.3. Effect of Temperature

The preparation of the silver nanoparticles was also monitored by variations in the temperature of 30 °C, 50 °C, and 70 °C on the constant concentration of extract and silver nitrate.

### 2.4. Antibacterial Activity of the Synthesized SNPs

Antibacterial activity of the synthesized SNPs from both fresh and dried plants was evaluated against pathogenic-resistant *Staphylococcus aureus* (ATCC 25923), *Bacillus subtilis* (ATCC 6633), *Escherichia coli* (ATCC 25922), and *Streptococcus pneumoniae* (ATCC 6305) by the Agar Well Diffusion Method (for details, see the supplementary data). The zone of inhibition was measured at different concentrations of AgNPs (20, 40, 80, and 100 µg/mL) after 24 h of incubation at 37 °C. The MIC (minimum inhibitory concentration) was calculated by comparison with Imipenem as a positive control and DMSO as a negative control [47,48].

### 2.5. Photocatalytic Degradation Activity of the Biosynthesized SNPs

The photocatalytic activity of the biosynthesized SNPs was evaluated by degradation of methylene blue under sunlight irradiation. Next, 1 mg of TSDESNPs (*T. Stocksianum* dry plant extract silver nanoparticles) was added to 10 mL of MB solutions (1 mg/100 mL) and allowed to stir under dark conditions. After 30 min, the solution was exposed to sunlight, and photocatalytic degradation was monitored after time intervals of 1 to 3 h by UV spectrophotometer. Pure methylene blue solution of the same concentration was taken as a control.

## 3. Results and Discussion

**Scheme 1:**
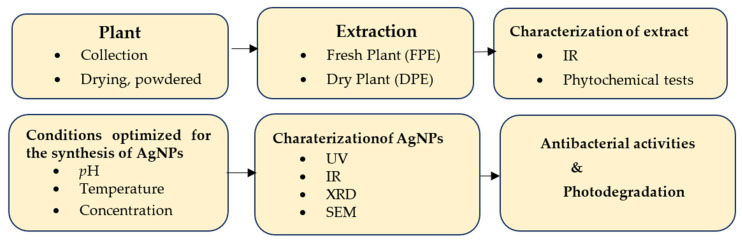
Scheme of study for the biogenic synthesis of the silver nanoparticles by *Teucriumstocksianum*.

A systematic scheme of studies was designed for the biogenic synthesis of the silver nanoparticles by using *Teucrium stocksianum* extract (Scheme 1). Initially, different classes of compounds present in the plant extract were detected by standard tests. These compounds have their role in the process of bio-reduction, capping, and stabilizing of the nanoparticles. Fresh and dried plant extracts were separately prepared for the detailed study and conditions were optimized by varying the physiochemical conditions (concentration, temperature, and pH). Extensive characterization techniques, including UV–Vis spectroscopy, FT-IR, SEM, DLS, and XRD analyses were employed to study the synthesis and properties of the prepared SNPs. Biological applications of the synthesized SNPs were demonstrated against multi-drug-resistant (MDR) pathogens. Moreover, the photocatalytic ability of the nanoparticles was evaluated by degradation of the methylene blue dye under sunlight.

### 3.1. Preliminary Phytochemical Screening of the Teucrium stocksianum

Phytochemical screening was carried out to identify different classes of natural products in the *Teucrium stocksianum* crude extract. Results revealed (Table 1) that alkaloids, tannins, saponins, sterols, and flavonoids are present in the plant crude extract (Figure S1), which may be responsible for the bioreduction, capping, and stabilization process.

### 3.2. Synthesis of the Silver Nanoparticles Using Teucrium stocksianum

The preliminary identification of crude extract helped to deduce that the plant extract had a significant potential to act as bioreducing media for the synthesis and stabilization of the metal nanoparticles. Therefore, we started our studies to optimize the condition by varying different physio-chemical parameters that include concentration, temperature, and pH. The synthesis of SNPs was initially identified by visual observation, where the color of the reaction mixture carrying *Teucrium stocksianum* extract and AgNO_3_ was turned from watery yellow to dark brown on the formation of silver nanoparticles. The dark brown color is the characteristic of silver nanoparticles due to the size-dependent surface plasmon resonance of AgNPs.

### 3.3. UV–Vis Analysis of Silver Nanoparticles

In order to study the process of reduction of silver ions to silver NPs by plant extract, UV–Vis spectroscopy was used. This technique was used to monitor the reaction process and to observe the optical properties of prepared NPs. Wavelength vs. Absorption spectra were recorded in the range of 350 nm to 800 nm. The absorbance bands were observed between 415 nm and 575 nm depending upon the reaction conditions. These bands are due to surface plasmon resonance caused by the excitation of valence electrons. Some factors affect the intensity of absorption band, which are discussed below.

#### 3.3.1. Effect of Concentration

**Figure 1:**
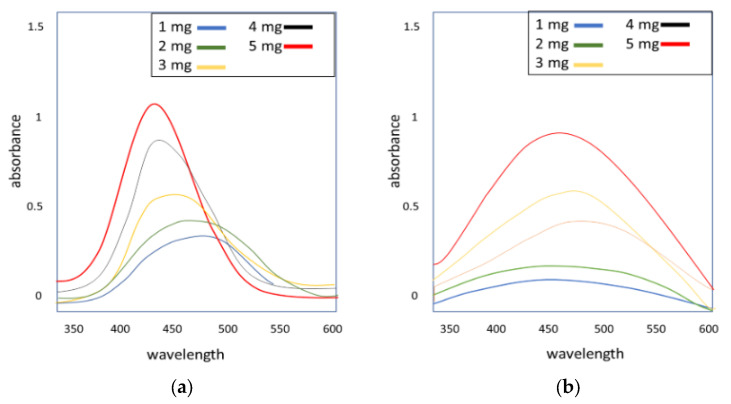
Effect of extract concentration on the preparation of SNPs (**a**) for TSADE and (**b**) for TSFPE.

Extract concentration was changed from 1 mg/1 mL to 5 mg/1 mL. It was observed that an increase in the extract concentration increases the rate of silver NPs’ formation and influences their size. With an increase in extract concentration, a blue shift from 470 to 430 nm indicated that the mean diameter of NPs decreased. Sharp UV–Vis peaks were observed on increasing the extract concentration. This indicated nanoparticles with smaller size because, at a higher extract concentration, more biomolecules are available that protects the silver NPs from aggregation, while lower extract concentration could reduce the silver ions but could not protect them from accumulation due to the low concentration of bioactive molecules responsible for capping the silver NPs. Broad peaks were observed in the case of silver NPs’ synthesis from TSFPE (*T. Stocksianum* fresh plant extract) which indicated large and poly disperse NPs, while sharp peaks were observed in the case of TSADE (*T. Stocksianum* fresh plant extract) (Figure 1).

#### 3.3.2. Effect of pH

**Figure 2:**
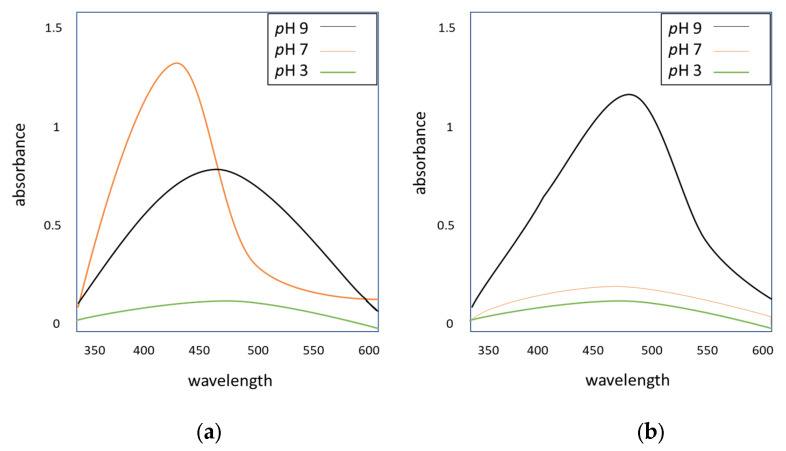
Effect of pH on the preparation of SNPs (**a**) for TSADE and (**b**) for TSFPE.

Literature reveals that pH also plays a key role in determining the shape, morphology, optical properties, and size of nanoparticles (NPs) synthesized from the plant extract. Particle size can be controlled by changing the pH of the media. This influential role of the pH in determining the chemistry of NPs is attributed to the capability of pH to change the charges of bioactive molecules present in the plant extract, leading to changes in their reducing and capping potential. The result demonstrated that pH has a prominent effect on the bioreduction of silver metal by *Teucrium stocksianum.* For the dry plant extract (TSADE), absorption was observed at 430 nm at neutral pH, while a peak at 490 nm was observed at a pH of 9. On the other hand, for the fresh plant extract (TSFPE), no absorbance was observed at pH levels 3 and 7, whereas a peak at 471 nm was detected at pH level 9 (Figure 2). Hence, for TSFPE, alkaline pH (9) was found more suitable, whereas neutral and alkaline pH can both successfully provide the silver NPs. In the case of TSADE, pH 7 was found the optimal where the intensity of the brown color was not changed after 12 h, or even after being kept for a week, indicating that stable silver nanoparticles are formed. Similarly, for the TSFPE extract, stable nanoparticles were obtained at pH 9 and, after 14 h, no further change in the intensity of brown color was observed.

#### 3.3.3. Effect of Temperature

**Figure 3:**
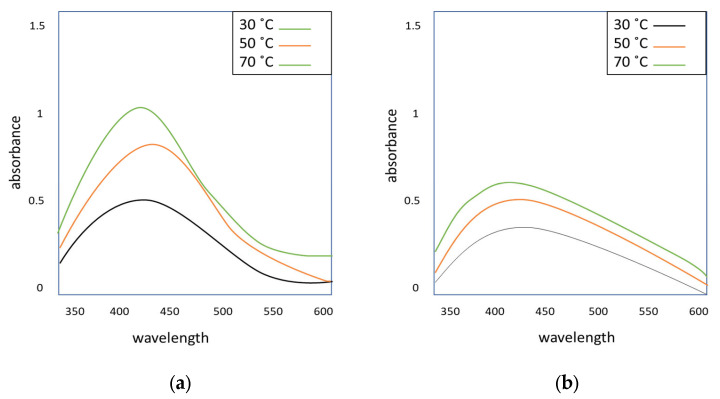
Effect of temperature on the preparation of SNPs (**a**) for TSADE and (**b**) for TSFPE.

Temperature variation was evaluated by maintaining the temperature of the reaction mixture at 30 °C, 50 °C, and 70 °C (Figure 3). An increase in temperature leads to a rapid reduction of silver as observed by UV spectroscopy. It is generally considered that an increase in temperature promotes homogeneous nucleation allowing the formation of silver NPs of small size. Sharp narrow peaks appeared with the rise in temperature in lower wavelength regions clearly indicating the smaller size NPs of silver. For TSFPE, a decrease in λ_max_ was noted with the rise in temperature, but the peaks were broader than dried air extract. In recent studies, we observed that 70 °C is the optimum temperature for silver NPs formation.

After monitoring the effects of various factors, i.e., plant extract concentration, pH, and temperature, the results revealed that the best-optimized conditions for TSDESNPs are a pH 7, at 70 °C, with 5 mg/1 mL concentration.

### 3.4. Characterization of the Synthesized Silver Nanoparticles

The UV–Vis spectroscopy clearly indicated the successful synthesis of silver nanoparticles with an aqueous extract of *Teucrium stocksianum*. The IR, DLS, SEM, and XRD analyses further helped to characterize the nature, size, and morphology of the synthesized silver nanoparticles (SNPs).

#### 3.4.1. FT-IR Analysis of the Plant Extracts and Silver NPs

**Figure 4:**
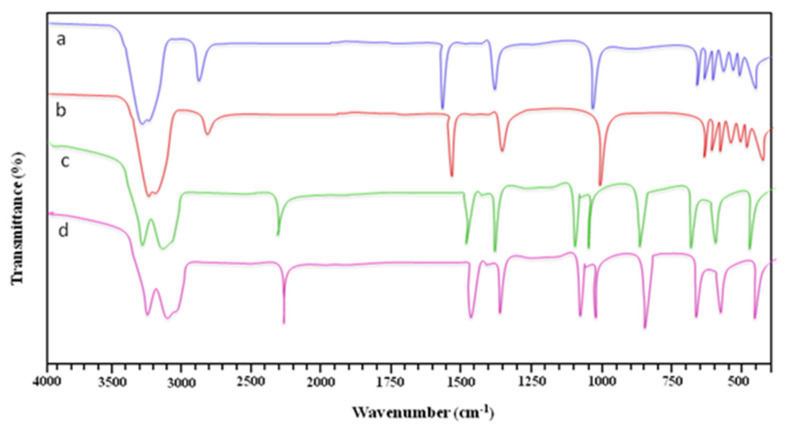
FT-IR spectra (**a**) TSADE, (**b**) TSDESNPs, (**c**) TSFPE, (**d**) TSFESNPs.

The FT-IR helped to identify the functional groups present in constituents of the plant extracts which may be involved in the synthesis and stabilization of the silver nanoparticles. These functional groups can play dual roles in green synthesis, acting as reducing as well as capping agents. A comparison of the FT-IR spectra of the fresh and dried plant extract and the silver nanoparticles prepared thereof is displayed in Figure 4. AnIR band is observed at 3267.41 and 3248.13, corresponding to OH stretching of alcoholic or phenolic groups. A peak was found at 2927.92 cm^−1^ corresponding to C−H stretching. Peaks at 1604.77 and 1400.32 cm^−1^ are the characteristic peaks of C=O and C−C stretching vibrations of aromatic compounds, respectively. These are the major peaks of different functional groups found in the dried extract, as shown in Figure 4a.

In the FT-IR spectrum of TSDESNPs, there is a change in these peaks which appeared at 3226.55 and 3210.45, shown in Figure 4b, which is a clear indication of the involvement of the -OH group in the NPs’ synthesis. Another prominent peak in the pure dry extract is 1697.36 which shifts to 1604.77 in TSDE silver NPs, which are the characteristic peaks of unsaturated ketones. Therefore, these two functional groups are considered to be involved in the reduction of silver ions and stabilization of the synthesized nanoparticles. Prominent peaks in the IR spectrum of TSFPE were observed at 1417.68, 1490.97, 3370.97, and 3282.84, as shown in Figure 4c. These peaks are attributed to aromatic compounds, carbonyl functional group, and hydroxyl stretching, respectively. Therefore, it is deduced that either polyphenols, alcoholic, carbonyl, and/or carboxylic groups present in the *Teucrium stocksianum* are responsible for the silver NPs’ formation.

#### 3.4.2. SEM Analysis of the Synthesized Silver NPs

**Figure 5:**
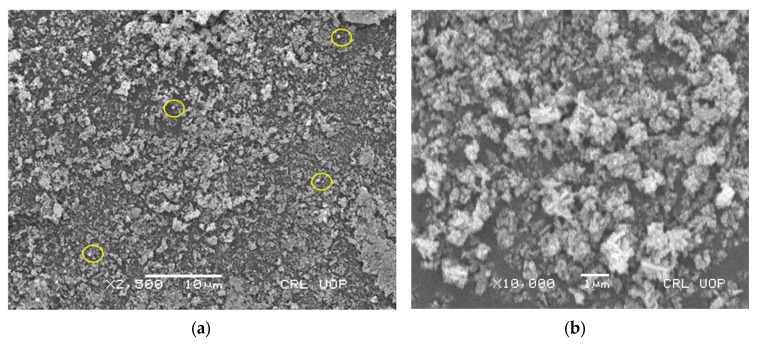
SEM analysis of the synthesized SNPs at different resolutions. (**a**) polydispersed. (**b**) coagulated.

The surface morphology and size of the silver NPs were evaluated by SEM (scanning electron microscope). The results displayed the synthesis of spherical silver NPs ranging in size from 67 nm to 90 nm, and of a polydispersed nature as highlighted in Figure 5a, while, at higher resolution, coagulation of silver NPs can be seen, shown in Figure 5b. The size of the NPs plays a vital role in their biological applications. It is considered that the rate of diffusion through biological membranes is highly dependent upon the size of particles. Reported literature revealed that small-size NPs are more effective due to more penetration power, but their too small size also enhanced the toxicity. Therefore, medium-sized nanoparticles are highly recommendable for the study of biological evolution [49,50]. These results were encouraging to evaluate the microbial activity of the synthesized nanoparticles against multi-drug-resistant pathogens.

#### 3.4.3. Powder X-ray Diffraction (PXRD) Analysis of the SNPs

**Figure 6:**
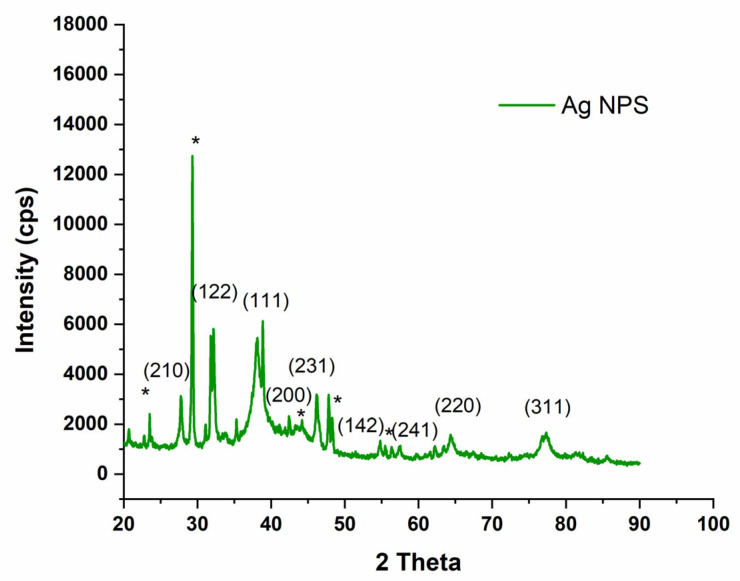
PXRD analysis of the synthesized SNPs.

The crystal structure and phase purity of the synthesized NPS were evaluated by XRD (X-ray diffraction). The XRD pattern of Ag NPS is shown in Figure 6. The diffraction peaks at 2θ values of 27.71°, 32.15°, 38.13°, 44.3°, 46.11°, 54.63°, 57.48°, 64.35°, and 77.40° corresponding to the reflection planes of (210), (122), (111), (200), (231), (142), (241), (220) and (311), respectively, indicating the FCC structure of Ag (JCPDS, file No.04-0783). The appearance of such sharp peaks warrants the formation of crystalline Ag NPS using *Teucrium stocksianum*. However, some additional unassigned peaks (asterisked in Figure 6) represented at the start may come from the crystallization of bio-organic moiety on the surface of Ag NPS as reported previously [51,52].

#### 3.4.4. DLS Analysis of the Synthesized SNPs

**Figure 7:**
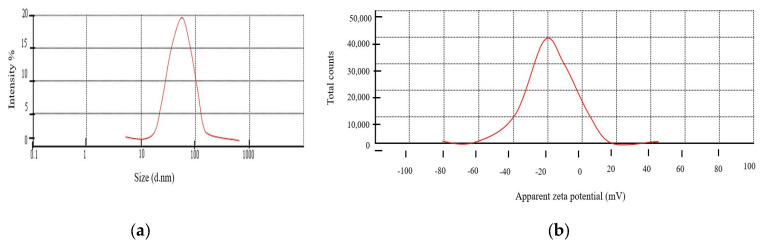
(**a**) Hydrodynamic size distribution. (**b**) Zeta potential (mV) of the synthesized silver nanoparticles.

Dynamic light scattering (DLS) analysis was employed to determine the hydrodynamic size and aggregation state of the synthesized nanoparticles. Results indicated (Figure 7a) that the particles were distributed in the size range of 1–120 nm, whereas a higher intensity was observed for the median size of 61 nm. The maximum total counts at −12 mV by zeta potential (Figure 7b) indicated that the synthesized SNPs are of good stability and colloidal properties due to the electrostatic repulsion. The negatively charged functional groups carrying electronegative atoms from the plant extract are responsible for significant capping to provide the colloidal stability of the SNPs.

### 3.5. Antimicrobial Activity of the Synthesized Silver NPs

Antimicrobial activities were studied against different drug-resistant pathogens at different concentrations. The results revealed that the biosynthesized SNPs exhibit high antimicrobial activity at low concentrations as compared to the parent plant extract. The MIC values were found between 5.3 µg/mL and 9.7 µg/mL for the biosynthesized SNPs, whereas the aqueous extract of the plant showed much high values of MIC, i.e., between 69 and 98 µg/mL (Table 2).

**Figure 8:**
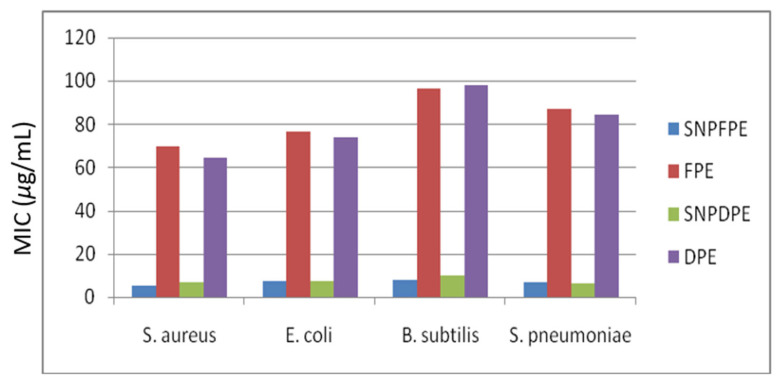
Comparison of the antimicrobial potential of the prepared SNPs with the parent plant extract.

Graphical representation (Figure 8) demonstrated that the antimicrobial potential of the prepared SNPs is very high in comparison to the respective plant extract.

### 3.6. Photocatalytic Activity of the Synthesized Silver NPs

**Figure 9:**
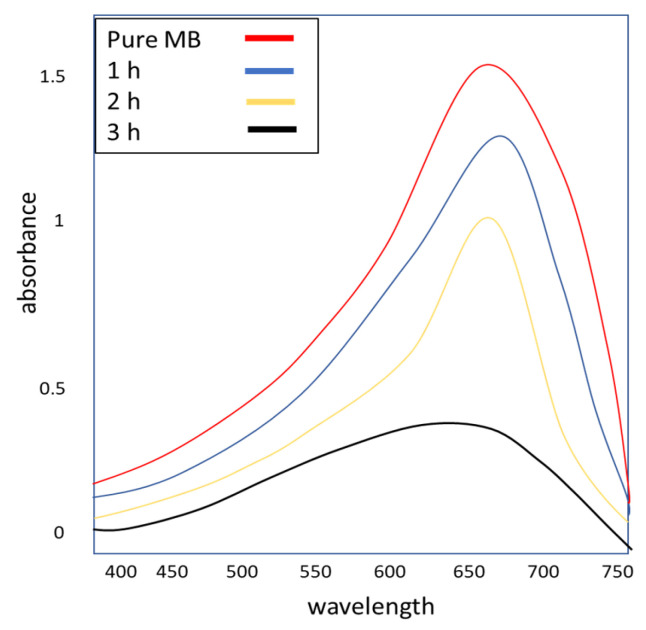
Photodegradation of the MB dye in the presence of the synthesized SNPs.

Synthetic dyes are commonly used in the paper and textiles, food, ink, and cosmetic industries. Methylene blue is commonly released through the textiles industries. It depletes oxygen from the water surface causing the death of flora and fauna in aquatic systems. It also causes toxic effects in humans; therefore, its degradation is highly required to control its harmful impact. Elimination of the organic waste from the ecosystem using silver NPs can provide a low-cost eco-friendly solution. The photocatalytic degradation of organic dyes is one of the important domains of SNPs’ utility. Methylene blue is an aromatic heterocyclic compound mostly used in textiles as a dye. The photocatalytic activity of biosynthesized silver NPs was evaluated by degradation of methylene blue (MB) under sunlight irradiation. A gradual fading from blue to a colorless solution under sunlight indicated that the dye degradation started within an hour. The decrease in the characteristic absorption of MB dye at 667 nm was monitored at different time scales, where a rapid decrease was observed after 3 h exposure (Figure 9).

## 4. Conclusions

In conclusion, the bioreducing potential of the *Teucrium stocksianum* was evaluated in detail on different physiochemical parameters. Biosynthesized silver nanoparticles were characterized by crystalline nature with a spherical shape and in the size range of 1–120 nm, whereas the median size was observed at 61 nm. The morphology, physiochemical properties, and stability of the synthesized nanoparticles were in agreement to serve as antimicrobial agents. Biological evaluation deduced their significant biological potential against multi-drug-resistant (MDR) pathogens. The synthesized silver nanoparticles were found many-fold more active (MIC = 5.3 µg/mL–9.7 µg/mL) in comparison to their parent fresh plant extract (MIC = 69–98 µg/mL). Moreover, the prepared nanoparticles exhibited the efficient potential to degrade the MB dye within 3 h of sunlight exposure.

## Figures and Tables

**Table 1:** Detection of different classes of natural products in *Teucrium stocksianum*.

No.	Class	Test	Reagent	Observation	Result
1	Alkaloids	Hager’s Reagent	A saturated solution of picric acid	Yellow precipitates	Positive
2	Tannins	Braymer’s reagent	5% FeCl_3_	Dark green color	Positive
3	Sterols	Salkowski’s reagent	Conc. H_2_SO_4_	Red-colored ring formation	Positive
4	Flavonoids	Alkaline reagent	2% NaOH + drops of HCl	The yellow color disappears with the addition of HCl	Positive
5	Saponins	Foam Test	Distilled water	Formation of foam	Positive
6	Waxes	Saponification	Methanolic NaOH	No saponification	Negative

**Table 2:** Minimum inhibitory concentration of silver NPs and parent plant extracts on the microbicidal viability.

	MIC (µg/mL)			
Microbes	SNPFPE	FPE	SNPDPE	DPE
*Staphylococcus aureus*	5.3	69.5	6.8	64.7
*Escherichia coli*	7.4	76.7	7.1	73.9
*Bacillus subtilis*	8.1	96.5	9.7	98.2
*Streptococcus pneumoniae*	6.7	86.9	6.3	84.3

## Data Availability

All the data are contained in the manuscript.

## Supplementary Materials

The following supporting information can be downloaded at: https://www.mdpi.com/article/10.3390/nano13081343/s1, Figure S1: Identification test for different natural product classes in *Teucrium strocksinum*.
