# Supplementary material for: Green Synthesis: The Antibacterial and Photocatalytic Potential of Silver Nanoparticles Using Extract of Teucrium stocksianum
Source: Nanomaterials (Basel). 2023 Apr 12;13(8):1343. doi: 10.3390/nano13081343 (PMC10145101; doi:10.3390/nano13081343)
Supplement: Supplementary file 1 [file nanomaterials-13-01343-s001.zip › nanomaterials-2290697-supplementary.pdf]

nanomaterials

MDPI

Supplementary Information

Green Synthesis: The Antibacterial and Photocatalytic Potential of Silver Nanoparticles Using Extract of *Teucrium stocksianum*

Iqra Rehman[1],Humaira Yasmeen Gondal[1,*], Roshan Zamir[1],Sami A. Al-Hussain[2],Fozia Batool[1],Ali Irfan[3], Sobia Noreen[1], TaleehaRoheen[1], Muhammad Nisar[1]andMagdi E. A. Zaki[2,*]

1 Institute of Chemistry, University of Sargodha, Sargodha40100, Pakistan; iqrarehman0042@gmail.com (I.R.); zamirg786@gmail.com (R.Z.);fozia.batool@uos.edu.pk (F.B.); sobia.noreen@uos.edu.pk (S.N.); taleeha.rohin@uos.edu.pk(T.R.); nisarbutt88@gmail.com (M.N.)
2 Department of Chemistry, College of Science, Imam Mohammad Ibn Saud Islamic University (IMSIU), Riyadh 13623, Saudi Arabia; sahussain@imamu.edu.sa
3 Department of Chemistry, Government College University Faisalabad, Faisalabad 38000,Pakistan; raialiirfan@gmail.com
* Correspondence: humaira.yasmeen@uos.edu.pk(H.Y.G.);mezaki@imamu.edu.sa(M.E.A.Z.)

Antibacterial activity

Agar-well diffusion method

The agar well diffusion method [47] was applied to investigate the antibacterial activities of plant extract and the synthesized silver nanoparticles at different concentrations of 20, 40, 80, and 100µg/ml. The McFarland standard (0.5 equivalents) was prepared to adjust the turbidity of the target bacterial suspension. A cup-borer well was prepared in the Mueller-Hinton agar plates, and 0.1 mL of the suspension of the tested bacteria was pipette in the well. The plates were kept at room temperature for 1 h, in the refrigerator for 3 h, and then incubated at 37 °C for 24 h. Imipenem and DMSO were used as positive and negative controls, respectively. The zone of inhibition was measured in triplicate by repeating the process.

The minimum inhibitory concentration (MIC)

The microdilution method was employed to evaluate the minimum inhibitory concentration (MIC) [48]. 96-well microtiter plates were prepared by dispensing the 95 µL of nutrient broth and inocula into each well. 100 µL of aliquot was separately prepared from the stock solutions of the samples and added into the wells. Further, 100 µL from the prepared serial dilutions was added into seven consecutive wells. The nutrient broth was added to one well, where distilled water and DMSO were used as positive and negative controls. Finally, the test plate was incubated at 37 °C for 24 h, and the bacterial growth was evaluated by absorbing at 620 nm on an ELISA reader. Minimum inhibition concentration (MIC) was determined as the lowest concentration of the extracts or NPs where no visible growth of bacteria was observed.

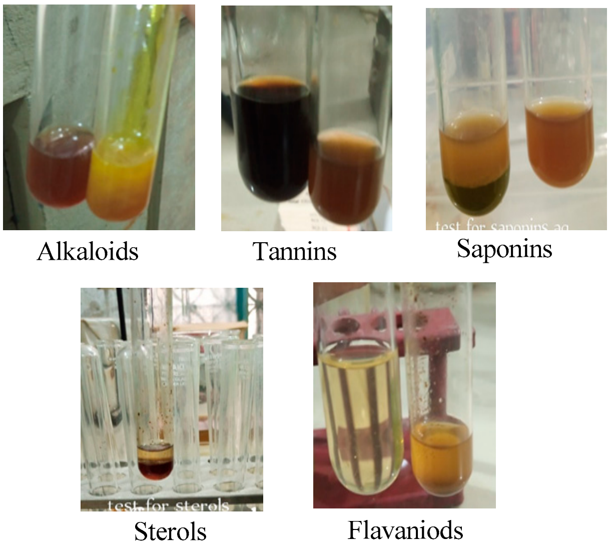

Figure S1. Identification test for different natural product classes in *Teucrium strocksinum.*

References

47. Perez, C. Antibiotic assay by agar-well diffusion method. Acta Biol. Med. Exp. **1990**, 15, 113–115.

48. bakht Dalir, S.J.; Djahaniani, H.; Nabati, F.; Hekmati, M. Characterization and the evaluation of antimicrobial activities of silver nanoparticles biosynthesized from Carya illinoinensis leaf extract. Heliyon **2020**, 6, e03624.
